# Lived experiences of adolescent girls following child sexual abuse: disclosure, coping, and perceived school support

**DOI:** 10.3389/fpsyt.2026.1895004

**Published:** 2026-10-22

**Authors:** Silvia S. V., J. Balamurugan

**Affiliations:** Department of Social sciences, School of Social sciences & Languages, Vellore Institute of Technology, Vellore, India

**Keywords:** adolescents, child sexual abuse, disclosure, female, perception, phenomenology, qualitative study, resilience

## Abstract

**Background:**

While research has established the adverse consequences of child sexual abuse (CSA), there remains a critical need to understand how adolescents process and make sense of their experiences, particularly within the Indian context, where discussions around sexuality and abuse often remain taboo.

**Objective:**

This study aimed to explore the perceptions of adolescent girls who have experienced CSA and to identify school-based strategies for prevention and intervention.

**Participants and setting:**

Seven adolescent girls aged 14–18 years were purposively selected from high and higher secondary schools in a district of Tamil Nadu, India. The participants were identified through a screening process following CSA awareness sessions attended by 517 students.

**Methods:**

A qualitative approach using semi-structured interviews and thematic analysis was employed to explore participants’ perceptions and experiences. Data were analysed using ATLAS.ti to identify key themes. Thematic saturation was achieved by the seventh interview, with no substantively new themes emerging during the final stage of analysis.

**Results:**

Four major themes emerged: (1) Narrating and Disclosing CSA as a Continuum of Expression, (2) Post-Traumatic Navigation: Inner Worlds and Outer Responses, (3) Perceptions of Perpetrators and Sociocultural Influences, and (4) Cultivating Safe Havens: Schools as Engines of Prevention and Change.

**Conclusion:**

Adolescents’ experiences of CSA were deeply influenced by emotional readiness, social context, and cultural norms. Despite their experiences of abuse, participants described the moments of resilience and highlighted the role of schools in fostering awareness, emotional support, and safe avenues for disclosure. The study underscores the need for systemic and empathetic interventions that center survivors’ voices and strengthen school-based prevention and support.

## 1 Background

Child sexual abuse (CSA) is recognized as a major public health concern and a form of Adverse Childhood Experience (ACE), with potentially long-lasting effects on health and overall well-being (1). Children who experience sexual abuse are at increased risk of psychological difficulties both immediately and over time (2). Research has consistently documented internalizing difficulties such as anxiety and depression, as well as externalizing behaviors such as aggression and defiance, together with symptoms of post-traumatic stress and dissociation (3–5). More recent research continues to demonstrate that the consequences of CSA are not uniform and that children may experience different psychological and social trajectories following abuse. Recent qualitative evidence has particularly highlighted the possibility of resilience and the importance of protective factors in shaping recovery following CSA (6, 7).

With CSA remaining a significant concern in India, it is important to understand not only its psychological consequences but also how children interpret, disclose, and navigate their experiences within their social environments (8). Kacker et al. (9), India's sociocultural context, marked by social inequality, gender-based discrimination, and persistent stigma surrounding sexuality, may influence both the experience of abuse and children's willingness to disclose it (10). Traditional social structures that emphasize obedience to elders and discourage open discussion of sexuality may further restrict children’s opportunities to speak about abuse (11–13). Although India enacted the Protection of Children from Sexual Offences (POCSO) Act in 2012 to provide a comprehensive legal framework for protecting children from sexual offences, CSA continues to be associated with considerable stigma and underreporting (14).

Disclosure is particularly important because it can determine whether children receive protection, emotional support, and access to appropriate services. Earlier qualitative research has shown that disclosure is often a gradual process shaped by fear, shame, relationships with others, and expectations of how adults may respond. Evidence also indicates that adolescents may initially disclose to peers or trusted individuals rather than directly to formal authorities (15). These patterns remain relevant to contemporary understandings of CSA because disclosure should not be treated as a single event but as a process influenced by interpersonal and social contexts. Sanjeevi et al. (16), Understanding children's own accounts of why they disclose, remain silent, or selectively share their experiences is therefore essential for developing responsive support systems.

Schools represent an important setting for identifying and responding to children affected by trauma because they provide sustained contact with children and can potentially offer accessible pathways to support. Recent literature has increasingly emphasized the development of trauma-informed school environments in which staff recognize the effects of trauma, respond sensitively to students’ needs, and reduce practices that may contribute to traumatization. A 2024 scoping review identified three broad components of trauma-informed schools: organizational and policy reform, workforce training, and educational or clinical practices (Onipede et al., 2024). Recent reviews have also examined trauma-sensitive approaches within school settings, highlighting the importance of creating supportive environments rather than relying only on individual-level interventions (Lembke et al., 2024).

The role of schools is particularly relevant to children’s mental health. Recent evidence indicates that universal school-based interventions can improve emotional outcomes among children and young people, supporting the value of schools as settings for mental health promotion and early support (Hayes et al., 2025). Similarly, recent systematic evidence on school-based CSA prevention programs suggests that schools can provide an important context for teaching children about recognizing unsafe situations, personal boundaries, and appropriate responses to potential abuse (17). However, prevention and awareness alone may be insufficient for children who have already experienced abuse. Schools also need to provide safe and supportive pathways through which children can disclose their experiences and access appropriate psychological and protective services.

Shoib et al. (18) found that, Along side school-based support, resilience has received increasing attention in recent CSA research. Resilience should not be understood as the absence of psychological distress or as an expectation that children should simply "overcome" abuse. Rather, recent qualitative research conceptualises resilience in relation to the processes and protective conditions through which individuals maintain or regain functioning despite adversity. A 2023 systematic review of qualitative studies identified interpersonal relationships, personal agency, meaning-making, and supportive environments as important elements in the lived experience of resilience following childhood sexual abuse. More recent qualitative research with children who experienced sexual abuse similarly highlights the importance of supportive relationships, personal strength, and the ability to respond actively to adversity (7). These findings suggest that children’s responses to CSA should be understood within the interaction between individual capacities and the social environments surrounding them.

Children who experience abuse may internalize the experience, question themselves and their bodies, and develop feelings of responsibility or shame. Such responses can contribute to continuing emotional distress (8). Non-disclosure may further restrict access to protection and support and may contribute to continued vulnerability (19). In culturally conservative settings where discussions of sexuality remain difficult, these processes may be particularly complex. Finkelhor and Ormrod (20) found that understanding how adolescents interpret their experiences, negotiate disclosure, respond to perpetrators, and perceive available sources of support is therefore important for developing culturally responsive interventions.

Given that childhood and adolescence are important developmental stages, understanding children’s own perspectives is essential. Qualitative approaches are particularly valuable for exploring personal meanings and contextual experiences that may not be adequately captured through quantitative measures (21). Noll (22) states that listening to adolescents' accounts can help identify the gap between their needs and the support available to them and can inform the development of school-based prevention, disclosure, and mental-health support mechanisms. The present study therefore explores the lived experiences and perceptions of adolescent girls who have experienced CSA, with particular attention to disclosure, post-traumatic responses, sociocultural influences, and the perceived role of schools in prevention and support.

## 2 Aim and research questions

The aim of this study is to deepen our understanding and broaden our knowledge of children’s perceptions of child sexual abuse (CSA) based on their experiences. By doing so, the study aims to enhance prevention programs in evaluating and developing intervention programs tailored to assist female adolescent survivors of CSA in the Institution. By exploring these areas, the study aims to provide valuable insights that can inform and improve intervention strategies, ensuring that they are responsive to the needs and experiences of children.

Research Question: How do female school-aged survivors of child sexual abuse understand, express, and navigate their experiences, and what role do institutional environments, particularly schools, play in their coping and support?

## 3 Methods

### 3.1 Methodology

Employing a constructivist paradigm with a phenomenological approach, this study reports on individual’s lived experiences of CSA as the construction of subjective reality. Thus, an in-depth interview served as a primary source of providing phenomenal perspectives to explore the key objective.

### 3.2 Philosophical and theoretical influences

Qualitative research generates data that describe the ‘who, what, and where of events or experiences from a subjective perspective’ (23). From a philosophical perspective, this approach to research is best aligned with interpretative methods. These philosophical perspectives represent the view that reality exists within various contexts that are dynamic and perceived differently depending on the subject, therefore, reality is multiple and subjective (24).

### 3.3 Sampling strategy

The sexual abuse experienced by children included in the study was validated following the survey. Most of the children were abused by someone from the extended family (e.g., uncle, cousin). All the perpetrators were male. Children’s demographic information was collected using self-reported data, completed either independently or with a researcher, or support worker. The severity of Sexual Abuse (SA) was defined using the coding system outlined by Russell (25) were considered very severe involving physical contact with oral, vaginal, or anal penetration for most of the children.

Initially, the researchers engaged with 517 children under the age of 18 from a district in Tamil Nadu as part of a school-based awareness initiative on Child Sexual Abuse (CSA), its consequences, and prevention strategies. Following each awareness session, students were asked to complete a screening questionnaire designed to assess their understanding of CSA and its prevention. The questionnaire also included a sensitive, self-disclosure item asking whether the respondent had ever experienced any form of sexual abuse. If the response was “yes,” students had the option to indicate whether they were willing to share their experiences with the research team by marking an additional “yes.” These awareness sessions were conducted among students aged 14 to 18 years, with approximately 40 students attending from each class. From the overall group, seven adolescents who reported having experienced CSA and voluntarily expressed willingness to share their narratives were recruited for the study. The sampling strategy began with stratified sampling to systematically select schools across different strata within the district, ensuring varied representation. Subsequently, purposive sampling was employed to identify and select participants based on their self-disclosure and informed willingness to participate in the research.

This qualitative study relies on individual semi-structured interviews conducted with 7 adolescent girls who have experienced child sexual abuse. This study has chosen to adopt a qualitative method approach which will allow for the detailed description of adolescent girl’s personal reality and provide privileged access into their subjective experiences and perceptions.

A sample of 7 participants identified from schools by purposive sampling method in the one of the districts of Tamil Nadu, India. The participants were started with a single question: “You mentioned negative sexual experiences in the past. Can you talk to me about this?” This open-ended question did not use terms such as “rape” or “sexual violence” to allow participants to express themselves however they chose to describe their experience. Researchers used empathetic and cognitive reflections to accompany the participant’s narrative and encourage further development. Furthermore, those adolescent girls who required psychological assistance were referred to an appropriate provider. Findings in this paper stem from qualitative research interviews with children (aged 14 & 18 years) in Tamil Nadu, who had experienced sexual abuse when aged under 18 years. Interviews were designed and delivered in partnership with a specialist & child sexual abuse practitioner from the Suvar Organization (Name has been changed due to confidentiality), NGO addressing child abuse and maltreatment. Practitioner from NGO supported the research team to embed a trauma-informed approach to interviewing. This approach aimed to minimize potential for traumatization, which is often cited as a barrier to research with children on this topic.

### 3.4 Procedure

Upon first contact with the participants, the nature of the research project was explained and a date was selected to conduct an individual interview at the school (n = 7). Interviews, which lasted an average of 45 min, were conducted over the period of 3 months. Each interview was audio recorded to ensure accurate verbatim transcription. Participants were given a fictitious name allowing for the results to be analyzed with anonymity and confidentiality. All respondents and their parent/guardian gave their full consent before taking part in this study, which was approved by the [name deleted to maintain the integrity of the review process] Ethics Committee.

All potential participants, and their parents or carers, received verbal and written information about the research from project workers known to them. Study sought formal recorded consent (rather than assent) at the outset of all interviews with children. This was supported using age and competency adapted printed and verbal information, and where appropriate, support from practitioners to help children make informed choices about involvement.

#### 3.4.1 Sampling

Initially, 517 students aged 14–18 years participated in school-based awareness sessions on child sexual abuse and its consequences. Following the awareness sessions, students completed a screening questionnaire that included a self-disclosure item regarding experiences of sexual abuse. Students who indicated that they had experienced sexual abuse were also given the opportunity to express their willingness to share their experiences with the research team. Seven adolescents reported experiences of child sexual abuse and voluntarily expressed their willingness to participate in the study. These seven participants were subsequently recruited through purposive sampling based on the study’s inclusion criteria and their informed willingness to participate. By the seventh interview, no substantively new themes relevant to the research questions were identified, and the final interview provided further depth and confirmation of the emerging patterns. Recruitment was therefore concluded at seven participants.

The study does not treat the number of participants as a predetermined target. Rather, adequacy of the sample was considered in relation to the depth and richness of the participants’ narratives and the emergence of recurring patterns across interviews. Data saturation was assessed progressively during data collection and analysis by comparing each interview with previously analysed interviews and examining whether new codes, categories, or themes continued to emerge. By the seventh interview, the analysis indicated that no substantively new themes relevant to the research questions were emerging. The seventh interview therefore contributed further depth and confirmation to the patterns already identified rather than introducing a new thematic dimension. On this basis, recruitment was concluded with seven participants.

The decision to conclude recruitment was also consistent with the purposive and phenomenological nature of the study, which sought an in-depth understanding of the lived experiences of adolescent girls who had experienced child sexual abuse rather than statistical representation. The final dataset generated four overarching themes and their associated subthemes, providing sufficient depth to address the research objectives.

#### 3.4.2 Data saturation

Data saturation was assessed progressively throughout the data collection and analysis process. The researchers reviewed each interview alongside the preceding interviews to identify recurring patterns, codes, categories, and emerging themes. Data saturation is generally understood as the point at which further data collection no longer contributes substantively new information to the developing analysis (26, 27). As the interviews progressed, the accounts increasingly converged around the key areas explored in the study, including disclosure and expression of abuse, emotional and behavioral responses, perceptions of perpetrators, sociocultural influences, and the role of schools and other support systems.

By the seventh interview, the analysis was sufficiently developed, and no substantively new themes or categories relevant to the research questions were identified. The seventh interview provided further depth and confirmation of the patterns already identified rather than introducing new thematic dimensions. Therefore, recruitment was concluded at seven participants, as the data had reached thematic saturation within the defined study population and research context. This decision is consistent with qualitative research approaches in which sample adequacy is determined by the richness and informational contribution of the data rather than by a predetermined numerical requirement (28).

The decision to stop recruitment was also informed by the purposive nature of the study and its phenomenological orientation, which prioritized detailed exploration of participant’s lived experiences rather than numerical representation. The seven participants were adolescent girls aged 14–18 years who had self-disclosed experiences of child sexual abuse and voluntarily agreed to participate in individual, trauma-informed interviews. Given the sensitive nature of the topic and the depth of the interviews, each participant provided substantial narrative data for the development and refinement of the themes. The final dataset supported the identification of four overarching themes and their associated subthemes.

#### 3.4.3 Reflexivity

The researcher recognized that her personal, educational, and professional background could influence the research process and interpretation of participants’ accounts. She had studied in a government-aided school and had previously worked as a psychologist in an organization, where she had experience working with school-going children in school-based settings, including counselling-related roles. As a female researcher interviewing adolescent girls about experiences of child sexual abuse, she was also aware of the potential influence of her gender and prior experiences on the interview process.

Before data collection, the researcher was conscious of certain assumptions regarding child sexual abuse, particularly that it represents a significant public health concern, disproportionately affects girls, and is influenced by wider sociocultural and cultural factors. Recognizing these pre-existing understandings was important to avoid imposing them on participants’ narratives.

To minimize interviewer bias, the researcher engaged in reflexive practice throughout data collection and analysis. Following each interview, she documented her observations, immediate reflections, and personal responses to the participant’s narrative. She also reflected on her own assumptions and considered whether these had influenced the questions asked, the interpretation of participants’ accounts, or the identification of emerging elements within the data. This ongoing reflexive process helped the researcher distinguish between participants’ experiences and meanings and her own interpretations, while keeping the participants’ accounts central to the analysis.

#### 3.4.4 Trustworthiness

Several strategies were employed to strengthen the trustworthiness of the findings, particularly credibility, dependability, and confirmability. Member checking was used to enhance credibility by providing participants with an opportunity to review and clarify the researchers’ understanding of their accounts. This helped ensure that the interpretations remained consistent with the meanings conveyed by the participants.

Peer debriefing was conducted throughout the analysis process. The researchers discussed the developing codes, categories, and themes, questioned emerging interpretations, and considered alternative understandings of the participants’ narratives. These discussions helped to minimize the influence of a single researcher’s interpretation and contributed to the refinement of the final thematic structure. This is consistent with the study’s existing procedure in which two researchers collaborated to review and refine emerging concepts.

Reflexive memo writing was maintained throughout the data collection and analysis process. Following interviews, the researcher documented observations, initial interpretations, personal responses, and potential assumptions that could influence subsequent analysis. These reflexive notes were revisited during the coding and theme-development stages to distinguish participants’ accounts from the researcher’s interpretations and thereby support confirmability.

Dependability was supported through the systematic progression from interview transcripts to codes, categories, subthemes, and overarching themes. The researchers repeatedly reviewed the developing analysis and discussed the consistency and relevance of the emerging themes in relation to the original narratives.

Transferability was addressed by providing contextual and participant information relevant to understanding the findings while maintaining confidentiality. The study does not seek statistical generalization; rather, the detailed description of the participants, research context, and thematic findings allows readers to assess the potential relevance of the findings to other contexts.

#### 3.4.5 Participant characteristics

Relevant participant characteristics were documented to provide contextual information for interpreting the findings. These included educational grade, rural/urban residence, age at the time of abuse, time elapsed since the abuse, disclosure status, and counselling history. Counselling support was continuously provided to the participants by the NGO throughout the study period. This ongoing support was considered as part of the participants’ contextual background when interpreting their narratives, while maintaining confidentiality and avoiding the inclusion of personally identifying information.

#### 3.4.6 Interview transcription and translation

The interviews were conducted primarily in Tamil, the participants’ preferred language, to enable them to express their experiences comfortably and in their own words. Each interview was audio-recorded with the participants’ and their parents’/guardians’ consent and subsequently transcribed verbatim. The Tamil transcripts were then translated into English by the researcher for the purposes of analysis and presentation in the manuscript. During translation, particular attention was given to preserving the meaning, emotional tone, and contextual meaning of the participants’ expressions rather than translating the statements word-for-word. Where participants used colloquial expressions or culturally specific terms, their intended meaning was retained as closely as possible in the English translation.

The original Tamil transcripts were retained alongside the English translations and were referred to during the coding and interpretation process to ensure that the translated quotations remained consistent with the participants’ original accounts. The quotations presented in the manuscript are therefore English translations of the participants’ original statements.

### 3.5 Trauma informed interview approach

Interviews were conducted on a one-to-one basis by members of the research team and conceived as ‘guided conversations’ (24). The interview process was developed with a consideration of the potential impact of abuse and sought to maximize participant wellbeing and control throughout. They were designed using trauma-informed principles (29). Examples of how this commitment translated into practice included: the opportunity for participants to have a supporter present if helpful; the use of ‘stop’, ‘go’, and ‘pause’ cards to help participants indicate their need for a break; and the provision of a potential distractions like grounding strategies, or relaxation aids. After the interview, participants were offered short-time counseling or a referral to a support service for victims of sexual assault to access psychosocial support if needed.

The research employed an interview schedule that focused on several key areas: Recognition, Identification, and Disclosure, Help-Seeking and Support, Contact with Services.

#### 3.5.1 Interview questions

1. Would you like to talk about anything that happened to you that made you feel uncomfortable or unsafe? Only if you feel okay to share.
2. Can you tell me a bit about what was happening in your life around that time? Like at home or school?
3. Why do you think things like this (CSA) happen to children?
4. What do you think makes it hard for children to tell someone when something bad like this (CSA) happens?
5. How do you feel about talking to people like your parents, teachers, or friends if something like this (CSA) happens?
6. What comes to your mind when you think about the person who did this? How do you feel about them now?
7. What do you think schools, adults, or your community can do to make children feel safer and more protected?

### 3.6 Data collection and analysis

Sociodemographic information—including the child’s age, the participant’s status, the nature of sexual abuse experienced, and the relationship between the child and the perpetrator—was gathered through a questionnaire filled out by the female adolescents. To address the core research questions, a semi-structured interview guide was crafted, drawing from both empirical studies and theoretical frameworks surrounding perceptions of child sexual abuse and lived experiences. The interviews primarily explored children’s emotional and behavioral responses, as well as the actions they took following the abuse.

An inductive thematic analysis was employed, following the methodology outlined by Clarke and Braun (30). This process unfolded in three key phases: Coding – The data were broken down using a flexible coding framework informed by existing literature and emerging insights from the participants’ narratives, enabling the creation of both pre-defined and novel codes. Categorization – The coded excerpts were systematically reviewed and grouped to identify patterns and form sub-themes. Linking – Relationships between sub-themes were analyzed to generate overarching themes that captured the essence of the participants’ experiences. The qualitative data were organized and analyzed using ATLAS.ti (version 7; Scientific Software Development, 2013). To enhance the trustworthiness of the analysis, two researchers collaborated closely, engaging in ongoing discussions to refine and agree upon the emerging concepts, thereby supporting inter-rater reliability.

### 3.7 Ethical considerations

The study was a part of a doctoral thesis and was approved by the Institutional Ethics Committee [VIT/IECH/2024/15 IECH/20 April 2024/13]. Since the sample included children, the different types of informed consent/assent forms (for children and their parents/guardian) were prepared and approved by the Institutional Ethics Committee. Further, standard operating procedure protocols for distress management (in case of any participant experiencing a trigger) and for implementation of the mandatory reporting of CSA in the context of this research (in case of disclosure by any minor participant) were developed. These protocols were recommended and approved by the Institutional Ethics Committee. This was done to align the study with the “Protection of Children Against Sexual Offences (POCSO) Act, 2012” of India (Ministry of Women and Child Development, 2013; https://wcd.nic.in). In accordance with the provisions of the Act, the identity of child survivors including details such as their names and places of residence must be kept strictly confidential. Therefore, the name of the district and the specific location of the study have been deliberately omitted to uphold the ethical and legal standards mandated by the Act.

## 4 Results and findings

The Socio demographic profile of the participants has given in the **Table 1**.

**Table 1:** Socio demographic profile of the participants.

Participants	Pseudonyms	Age	Educational grade	Mother’s occupation	Father’s occupation	Age at abuse	Time since abuse	Disclosure status	Rural/urban residence
P1	SS	16	11^th^	Homemaker	Driver	8	8 yrs	No	Rural
P2	SN	15	10^th^	Homemaker	Advocate	10	5 yrs	No	Urban
P3	HN	15	10th	Tailor	Tailor	8	7 yrs	No	Rural
P4	RA	16	11^th^	Homemaker	Driver	14	2 yrs	Yes	Rural
P5	AD	15	10^th^	Domestic Helper	Unemployed	12	3 yrs	No	Urban
P6	RI	17	12th	Homemaker	Daily wages	8	8 yrs	Yes	rural
P7	SD	16	11th	Homemaker	–	10	6 yrs	No	rural

The distribution of participants according to their relationship with the perpetrator and the severity category is presented in **Table 2**.

**Table 2:** Seriousness of child sexual abuse by relationship with the perpetrator.

Items	Very serious child sexual abuse	Serious child sexual abuse	Least serious sexual abuse
Uncle (P1)			**✔**
Stranger boy (P2)			**✔**
Uncle (P3)			**✔**
Brother (P4)			**✔**
Known boy (Mother’s friend’s son) (P5)		**✔**	
Uncle (P6)			**✔**
Uncle (P7)			**✔**

Based on Diana E. H. Russell’s seriousness of intrafamilial and extra familial child sexual abuse by Relationship with the Perpetrator.

For the purpose of categorizing the types of child sexual abuse reported by participants, the study followed Russell’s classification of sexual abuse severity. The categories used in the analysis were:

• Very serious sexual abuse: Completed or attempted vaginal, oral, or anal intercourse, including cunnilingus.

• Serious sexual abuse: Completed or attempted genital fondling, simulated intercourse, or digital penetration, whether forced or unforced.

• Least serious sexual abuse: Completed or attempted intentional sexual touching of the buttocks, thigh, leg, other body parts, clothed breasts or genitals, and kissing, whether forced or unforced.

The main themes of the study and their relationships with the subthemes that were identified are presented in the **Table 3**.

**Table 3:** Themes and subthemes.

S.No	Themes	Subthemes	Codes	Quotation
1	Narrating and Disclosing Child Sexual Abuse: A Continuum of Expression	Internal Narratives and Modes of Expression	touched my breasts, Impact after the incident occurred, touched my breasts, shocked	He touched my breasts. I just pushed him away and ran from the place." (P2) "He literally placed his private part on my private part." (P5) "My uncle touched my breasts. I was shocked when this happened." (P6)
		External Disclosure: Barriers and Enablers	Fear of consequences, fear of mother, Fear of consequences, fear of physical abuse from mother, fear leads non-disclosure	I had no idea what had happened … I thought, 'How can I report this problem to parents?' 'What if it became a big issue?' I was afraid of 'What if a fight occurs in my family because of this incident?' 'What if this comes into news channels?'" (P1)
2	Post - Traumatic Navigation: Inner Worlds and Outer Responses	Asserting Voice in Silenced Spaces	handled better when happened for the 2nd time, confident to handle CSA	“And, I told him ‘Uncle, do not do this and I did not like it too. Otherwise, I would tell it to my mother.’ I was confident enough to fight back those issues as I was 12 years old at that time. I have handled it better.” (P5)
		Mapping Trust Beyond the Trauma	Boy’s intentions, boys can understand girl's issues, girls are hesitant to share, lack of openness is a significant issue, boys encourage open communication about menstruation	“But still, there are good boys/men too. Since some boys have the bad intention to do it. They tend to sexually abuse others.” (P1) “One friend is there. He used to tell, ‘If you have stomach pain, you can definitely tell it to me and I’m a science student,’ and I tend to tell, ‘Yes, I will if I have any issues.” (P5)
		The Inherited Burden of Shame and Blame	virginity is for both boys and girls, keeping the virginity only in women's body, blame the girl	“I have listened to a speech. In that speech, it is mentioned that virginity is for both boys and girls, but we will only keep the virginity in the women’s body only.” (P5) “Nothing would happen. They would blame me and support his son.” (P2)
3	Child’s Perception of Perpetrators and Influencing Factors	When Familiar Faces Become Threats	avoid going to perpetrator's place, perpetrator might be relative, despite scolding, child avoids perpetrator's places, Perpetrators include teachers, relatives, or anyone	"If the perpetrator is a relative, then the child would not go to their homes, and they would get scolded for not going to relatives' homes by the mother. Still, they used to avoid going to the places of the perpetrator (tuition teacher, relatives, or whoever the perpetrator), even though they get bad names." (P5)
		The Mask of Normalcy	Experiences of sexual abuse bothered me, wondering about his behavior,	"I used to wonder how come he can talk/smile to me without any guilt. Everyone was talking with him nicely. I might think that, 'He has done a big mistake, and nobody knows about this. How strange is this?' He is just roaming around happily. He used to ask, ‘Are you going to school? Which standard?’ and act like nothing has happened." (P1)
		Blueprints of Blame: Media, Masculinity, and Misconduct	men don't have self-control, women have self-control	"They used to watch inappropriate videos. If they get high, they tend to do it. They do not have self-control, whereas women have self-control, even though they also have sexual feelings. Women usually know the consequences very well. Men may not analyze the consequences." (P5) "Just because he is a boy, no one is bothered about his mistakes. Like, whatever wrong things boys do to girls—no one is asking him about his mistakes. So those small mistakes change into bigger mistakes, like sexual abuse." (P2)
4	Cultivating Safe Havens: Schools as Engines of Prevention, Empowerment, and Change	Embedding CSA Awareness and Accountability Mechanisms in School Systems	Ensuring availability of responsible authorities, child sexual abuse need to be discussed	“There must be improvement in many aspects related to child sexual abuse. For example, government has appointed a person to maintain the school activities. Like, collector in any district would take care of those things. Likewise, to monitor these issues, there should be appointed a responsible person who will be available. Awareness to be given more. All girls need to be even more bold. For which, topics related to child sexual abuse need to be discussed. Hence, this needs to be included in syllabus.” (P1)
		Bridging the Gender Divide: Integrating Empathy Through Co-Education	Distancing increases attraction, same class foster cooperation between gender	“If both are distant, then attraction increases … If they are in the same class, they would have helped each other.” (P5)
		The Trust Continuum: Teachers as First Responders and Emotional Anchors	knowledge leads reporting, students need to know	“Teachers can also address the topics of sexual abuse. Hence, students become bold. Then the students might know what it is about. In schools, they have conducted yoga for wellbeing. So, they can allocate classes for these. For at least, two times a year related to sexual abuse awareness. In that, there should be strong statement need to be said that ‘if any girls undergone such sexual abuse, they must report it’ and this team should encourage students in reporting the abuse.” (P6)

### 4.1 Narrating and disclosing child sexual abuse: a continuum of expression

Adolescents articulated their experiences of child sexual abuse (CSA) through both verbal and non-verbal forms of communication. Their capacity to express these experiences was closely linked to their individual perceptions, emotional processing, and levels of awareness regarding CSA. Typically, the initial response to such incidents was a vague but distressing sense of discomfort or confusion. Even before fully understanding the nature or implications of what had occurred, many adolescents reported feeling helpless, trapped, and unable to react in a way that ensured their immediate safety.

Importantly, the adolescent’s narratives and choice of language were influenced by their developmental stage, exposure to information about CSA, and emotional readiness. This section explores how internal narratives and external disclosures of CSA formed a continuum of expression for adolescents, encompassing both direct and indirect articulations and varying disclosure outcomes.

#### 4.1.1 Internal narratives and modes of expression

Some adolescents demonstrated a capacity to verbalize their experiences using direct, unambiguous language. These narratives reflected a clearer understanding of the abusive behavior and a willingness to label it accurately, without diluting the severity of the act. The use of anatomically correct terms and action-oriented verbs suggests a degree of confidence and emotional clarity.

“He touched my breasts. I just pushed him away and ran from the place.” (P2)

“He literally placed his private part on my private part.” (P5)

“My uncle touched my breasts. I was shocked when this happened.” (P6)

Conversely, another group of adolescents verbalized their experiences through vague or indirect language, often using euphemisms or metaphors. These narratives were marked by hesitation, emotional discomfort, or fear of judgment and were common among those with limited awareness or lower confidence in discussing sexual topics openly.

“Then, when I was scrolling the gallery on his phone, I saw ‘weird videos’ and asked him ‘What’s this?’ He said, ‘You just look into it,’ and then told me, ‘You are seeing this but why can’t you see mine too?’ I said, ‘No, I will not see this. Please leave me.’ He replied, ‘It’s okay, you can see.’ I said, ‘NO.’ This is the only thing that happened to me.” (P1)

“He did bad touch and done whatever requires to form the baby.” (P3)

Such expressions reflect a complex internal negotiation between the need to disclose and the struggle to find socially or emotionally acceptable language.

#### 4.1.2 External disclosure: barriers and enablers

Disclosure patterns emerged as deeply personal decisions influenced by the adolescent’s perceived safety, emotional readiness, and social context. Many participants cited fear of family conflict, social stigma, or disbelief as reasons for non-disclosure.

“I had no idea what had happened … I thought, ‘How can I report this problem to parents?’ ‘What if it became a big issue?’ I was afraid of ‘What if a fight occurs in my family because of this incident?’ ‘What if this comes into news channels?’” (P1)

“Of course, I cannot tell it to anyone … I would think, ‘What would happen if this became a big issue?’ or ‘What if this issue became a problem for me?’ or ‘What if they are not allowing me to go to school and make me sit at home?’” (P2)

“My parents do not have the mind set to listen the experiences of sexual abuse … When I had those abusive experiences, I feared my mother … I thought that ‘What if she beats me once I disclose this?’” (P5)

In contrast, adolescents who perceived a supportive environment whether through family, friends, or educators were more likely to disclose their experiences.

“My home is my biggest support. That is why I can talk to you confidently. Since my family knows about this, I could get the confidence to talk.” (P6)

“These issues will get prevented or avoided if all are taking a step for a change … So, everyone should tell their sexual abuse experiences if they had one like me at least to anyone.” (P4)

The narratives highlight that disclosure is not a singular event but rather a dynamic process influenced by an adolescent’s internal world and external environment. The environment plays a crucial role, shaping their perception of support and safety. Those who receive support may develop a more positive outlook, feeling that the world is a safe and nurturing place, whereas those who lack support may experience persistent helplessness that lingers throughout their lives. Recognizing disclosure as a continuum from private internalization to open expression can foster more sensitive and effective interventions for CSA survivors.

### 4.2 Post-traumatic navigation: inner worlds and outer responses

Following the experience of child sexual abuse (CSA), female adolescents exhibited a range of psychological, emotional, and behavioral responses. These reactions were shaped by various factors, including personal resilience, access to support systems, cultural norms, and levels of awareness regarding abuse and gender roles. Three key sub-themes emerged in how adolescents processed and responded to their experiences: Asserting Voice in Silenced Spaces, Mapping Trust Beyond the Trauma, The Inherited Burden of Shame and Blame

#### 4.2.1 Asserting voice in silenced spaces

Many adolescents demonstrated resilience and agency in confronting the abuse. Their responses varied depending on their level of awareness, confidence, and the perceived availability of external support. Empowered by a sense of self-worth or a protective instinct, some children directly confronted their perpetrators or disclosed the abuse to trusted adults such as parents or teachers. These responses were often linked to a belief that action could lead to protection or justice.

“And, I told him ‘Uncle, do not do this and I did not like it too. Otherwise, I would tell it to my mother.’ I was confident enough to fight back those issues as I was 12 years old at that time. I have handled it better.” (P5)

“One day, I went to tell my mom that ‘Anna (Brother) is touching me inappropriately,’ and then amma was saying that ‘I’ll warn him, but don’t tell this to anybody, especially to Appa.’ My mom warned him too.” (P4)

These narratives highlight the children’s attempts to resist or report abuse, which not only served as immediate coping mechanisms but also empowered them in reclaiming control over the situation. However, responses from adults such as minimizing the incident or discouraging further disclosure had the potential to either validate or suppress the child’s efforts.

#### 4.2.2 Mapping trust beyond the trauma

Some adolescents demonstrated the capacity to cognitively separate the abusive incident from other aspects of their lives. This positive reframing allowed them to maintain trust in others, particularly men and boys, despite having experienced abuse. These adolescents did not allow the trauma to define their entire worldview; instead, they selectively differentiated between the perpetrator’s actions and the behaviors of others in their social environment.

This ability to compartmentalize harmful experiences is indicative of emotional resilience and may reflect the presence of protective factors such as supportive friendships, prior knowledge about CSA, and personal coping skills.

“But still, there are good boys/men too. Since some boys have the bad intention to do it. They tend to sexually abuse others.” (P1)

“One friend is there. He used to tell, ‘If you have stomach pain, you can definitely tell it to me and I’m a science student,’ and I tend to tell, ‘Yes, I will if I have any issues.’” (P5)

These statements reflect a conscious effort by the child to resist generalizing their negative experience to all males. This nuanced understanding contributes to a more balanced social and emotional development and illustrates the child’s ability to foster trust and retain social connections post-trauma.

#### 4.2.3 The inherited burden of shame and blame

A significant theme that emerged was the Inherited Burden of Shame and Blame. The adolescents’ narratives suggest that how deeply entrenched patriarchal values shaped their perceptions of self, blame, and identity in the aftermath of CSA. Many expressed feelings of shame, fear of being blamed, or even a wish to be born male, viewing femininity as a source of vulnerability or burden.

“I have listened to a speech. In that speech, it is mentioned that virginity is for both boys and girls, but we will only keep the virginity in the women’s body only.” (P5)

“Nothing would happen. They would blame me and support his son.” (P2)

“I would think that ‘why did I born?’ I could have born as a boy.” (P3)

These reflections expose the deeply gendered framework within which girls are often socialized. The societal tendency to assign responsibility or shame to the female victim, while absolving or protecting the male perpetrator, contributes to internal conflict and a distorted sense of self-worth among adolescent girls. This theme also underscores the need for gender-sensitive education and culturally responsive interventions that challenge harmful norms and empower girls to reclaim agency over their bodies and experiences.

### 4.3 Child’s perception of perpetrators and influencing factors

This theme explores how children interpret the identity, behavior, and motivations of their abusers, along with the sociocultural influences that shape these perceptions. The narratives reflect deep emotional responses, cognitive dissonance, and attempts to make sense of betrayal, apathy, and the normalization of abusive behavior in society.

#### 4.3.1 When familiar faces become threats

A striking and recurring theme in the narratives was the experience of abuse by individuals familiar to the children—relatives, teachers, or community members. The violation of trust by someone the child knows not only intensifies the trauma but also complicates their ability to disclose or process the incident. Many participants expressed disbelief and distress at the idea that someone once considered trustworthy could cause such harm.

The betrayal by a known person left children emotionally vulnerable and isolated, often unable to fully articulate or even understand what had occurred due to their developmental stage. Unlike overt forms of sexual violence, the grooming behavior employed in CSA subtly manipulates the child into compliance, making it harder for them to recognize the act as abusive and resist it.

One participant explained how trust was broken and how this might affect the daily life of adolescent who have experienced child sexual abuse:

“If the perpetrator is a relative, then the child would not go to their homes, and they would get scolded for not going to relatives’ homes by the mother. Still, they used to avoid going to the places of the perpetrator (tuition teacher, relatives, or whoever the perpetrator), even though they get bad names.” (P5)

This narrative underscores how familial and social expectations can pressure children into continuing interactions with perpetrators, further silencing them and worsening the psychological burden.

#### 4.3.2 The mask of normalcy

Participants also reflected on the disturbing emotional dissonance they experienced when abusers appeared to show no guilt or remorse. Perpetrators often continued their interactions with the child as if nothing had happened, which left victims confused, disillusioned, and emotionally conflicted.

Children struggled to understand how someone who had committed such a serious violation could act normally in public, maintaining a friendly demeanor and receiving social acceptance. This behavior left the victims questioning not just the morality of the abuser, but also society’s failure to recognize or confront such harm.

As one participant reflected:

“I used to wonder how come he can talk/smile to me without any guilt. Everyone was talking with him nicely. I might think that, ‘He has done a big mistake, and nobody knows about this. How strange is this?’ He is just roaming around happily. He used to ask, ‘Are you going to school? Which standard?’ and act like nothing has happened.” (P1)

This reaction illustrates how perpetrators often maintain a facade of normalcy, which further invalidates the child’s experience and fosters a deep sense of injustice and helplessness.

#### 4.3.3 Blueprints of blame: media, masculinity, and misconduct

Another key insight from the children’s narratives was the perceived influence of media and societal norms on the behavior of perpetrators. Participants shared the belief that exposure to inappropriate content in films or online media, along with poor role modeling within families, contributed to the normalization of harmful behaviors among boys.

Children sensed a double standard in how boys and girls were socialized—boys were often not held accountable for their behavior, while girls were expected to bear the burden of shame and silence. The participants noted how this imbalance reinforced a sense of entitlement among boys and further enabled abusive behavior.

Interviewer: *Why do you think boys are indulged in sexually abusive behavior?*

Participant: *“Ahh … that I could not tell properly. How can I say … they might have watched scenes from cinema or parents. So, they imitate the behavior on girls.”* (P4)

“They used to watch inappropriate videos. If they get high, they tend to do it. They do not have self-control, whereas women have self-control, even though they also have sexual feelings. Women usually know the consequences very well. Men may not analyze the consequences.” (P5)

“Just because he is a boy, no one is bothered about his mistakes. Like, whatever wrong things boys do to girls—no one is asking him about his mistakes. So those small mistakes change into bigger mistakes, like sexual abuse.” (P2)

These reflections illustrate a deeply gendered social narrative where male misconduct is excused or minimized, creating a permissive environment for abuse. The participants’ comments reflect an early understanding of how social and cultural scripts around masculinity and power contribute to the persistence of child sexual abuse.

### 4.4 Cultivating safe havens: schools as engines of prevention, empowerment, and change

This theme captures the role of schools not merely as institutions of learning, but as transformative ecosystems where the seeds of awareness, empathy, and resilience against Child Sexual Abuse (CSA) can be intentionally cultivated. Participants emphasized that schools must move beyond traditional academic boundaries to adopt a proactive, holistic approach—embedding CSA education within curriculum, nurturing gender sensitivity, and building trust-based support systems involving teachers, peers, and caregivers. Schools, they argued, are not passive settings but active agents in CSA prevention and intervention.

#### 4.4.1 Embedding CSA awareness and accountability mechanisms in school systems

Participants voiced a strong need for the structured inclusion of Child Sexual Abuse (CSA) education within school curriculum. They emphasized that institutionalizing this subject through formal policy would not only raise awareness but also reduce stigma and empower students to identify, resist, and report abusive behavior. Integrating CSA education into the syllabus was seen as a necessary step to legitimize these conversations and treat them with the same importance as core subjects like health or social science.

“Schools have kept social as separate subject till 10th standard. Like that, they can add sexual abuse as a topic.” (P1)

Beyond curriculum content, adolescents advocated for the appointment of specific individuals within school administrations to take responsibility for monitoring CSA-related concerns. They envisioned a role like a district-level officer who would ensure that protective measures are in place, and that students have someone accessible and accountable to approach when issues arise.

“There must be improvement in many aspects related to child sexual abuse. For example, government has appointed a person to maintain the school activities. Like, collector in any district would take care of those things. Likewise, to monitor these issues, there should be appointed a responsible person who will be available. Awareness to be given more. All girls need to be even more bold. For which, topics related to child sexual abuse need to be discussed. Hence, this needs to be included in syllabus.” (P1)

Participants also emphasized the importance of age-appropriate sex education. Starting around age 15, they believed students should be taught about gender dynamics, bodily autonomy, boundaries, and healthy relationships. This, they argued, would allow adolescents to better understand and protect themselves in complex interpersonal situations.

“Teachers/schools can demonstrate about the gender and the science of attraction toward each other. But they are not trying to teach about it. Many girls think that ‘If sexual abuse happened to them, they used to hurt their body and commit suicide as they think that the body is no longer for use.’ So, through learning, these things can be avoided. If boy tries to do anything even when they are in relationship, the girl will make caution about the consequences. For that, they both need sex education about it at least start by the age 15 years.” (P5)

By embedding CSA-related content in the formal school framework and appointing responsible figures for oversight, participants believed that schools could move beyond passive awareness to active monitoring and early intervention. This combination of education and accountability would both validate students’ experiences and offer them clear, trusted pathways for reporting abuse and seeking help.

#### 4.4.2 Bridging the gender divide: integrating empathy through co-education

Participants reflected critically on how rigid gender segregation in schools may contribute to unhealthy curiosity, miscommunication, and distorted perceptions between boys and girls. Rather than protecting students, this divide was seen as potentially counterproductive—heightening attraction through distance and reducing opportunities for natural peer learning, support, and understanding.

“If both are distant, then attraction increases … If they are in the same class, they would have helped each other.” (P5)

They argued that co-educational settings, when thoughtfully managed, could promote empathy, normalize respectful interactions, and cultivate mutual understanding of gender dynamics. Integrating genders in classroom settings was seen as not only educational but protective, allowing children to develop healthier relationships that could buffer against CSA risks.

Participants believed that inclusive classrooms would allow both genders to understand each other’s challenges, especially around sensitive topics like sexual abuse. They suggested that such integrated spaces would also encourage boys to act as allies or protectors rather than potential aggressors, while empowering girls to express themselves more freely without fear or stigma.

#### 4.4.3 The trust continuum: teachers as first responders and emotional anchors

Participants described the profound role that teachers could play—when emotionally available and appropriately trained—in creating safe spaces for disclosure. Teachers were viewed as crucial intermediaries between students and formal reporting mechanisms. However, this depended heavily on the teacher’s demeanor and willingness to listen without judgment.

“Teachers need to be friendly in nature instead of rude to their students. I do not know what actions teachers would take for the issue. But at least we can share if they are in a state to listen to us. In addition, teachers might guide the student in a healthy way and they would encourage the girl child to share this with their parents. If the girl has no confidence in discussing these things with parents, teachers would give the confidence to help her to talk with their parents. Then the child would report to parents.” (P1)

In addition to emotional support, participants proposed regular awareness programs within school settings. Just as physical well-being is addressed through activities like yoga, they believed emotional safety around CSA must also be institutionalized through periodic education, clear reporting encouragement, and direct statements about the importance of seeking help.

“Teachers can also address the topics of sexual abuse. Hence, students become bold. Then the students might know what it is about. In schools, they have conducted yoga for wellbeing. So, they can allocate classes for these. For at least, two times a year related to sexual abuse awareness. In that, there should be strong statement need to be said that ‘if any girls undergone such sexual abuse, they must report it’ and this team should encourage students in reporting the abuse.” (P6)

Participants envisioned teachers not only as conveyors of information but as emotional anchors who could help students navigate the psychological aftermath of CSA. They suggested that when teachers affirm students’ voices and provide safe disclosure channels, it can redefine the school space as a sanctuary for healing and action. The consistent presence of a trusted adult figure was seen as critical to enabling confidence in reporting and overcoming the shame often associated with such experience.

## 5 Discussion

This study explores the multifaceted and deeply personal ways in which adolescent girls experience, narrate, and respond to child sexual abuse (CSA), emphasizing the continuum of expression that shapes both internal meaning-making and external disclosure. The participant’s narratives suggest that expressions were shaped by their developmental stage, emotional readiness, and familiarity with the language of abuse. These expressions are broadly consistent with what Summit (31) conceptualized as part of the “Child Sexual Abuse Accommodation Syndrome,” where children struggle with secrecy, helplessness, and delayed disclosure. As Gomes (32) observed, narrative coherence in CSA disclosures is often shaped by the survivor’s cognitive and emotional ability to process and verbalize the abuse. This form of articulation was more prevalent among adolescents who had prior awareness of CSA or had received relevant education or support, echoing findings from Cohen’s (33), which emphasized the importance of age-appropriate, non-stigmatizing language in facilitating open discussion of abuse.

As Cohen’s (33) framework suggests, the use of softened or coded language serves as a protective linguistic barrier, insulating the child from the full emotional weight of their experience. McElvaney (34) and Morrison et al. (35) note that these fears are common among CSA survivors, often leading to significant delays in disclosure or silence. This finding is consistent with previous literature by Alaggia (36) and Morrison et al. (35), which highlights perceived social support as an important facilitator of CSA disclosure, helping children feel safer and more confident in speaking about their experiences. While some participants demonstrated the ability to articulate the abuse in clear, unambiguous terms, others relied on indirect language, euphemisms, or vague references. These differences suggest that disclosure is not a one-time event but a gradual and context-dependent process, often shaped by fear, uncertainty, or shame. This aligns with existing literature that positions disclosure as a complex, non-linear trajectory, rather than a singular moment of truth-telling.

The psychological aftermath of CSA among participants ranged from proactive resistance to deeply internalized blame and shame. Some adolescents described moments of resilience and self-efficacy by confronting perpetrators or confiding in trusted adults, underscoring their agency even in the face of trauma. However, others expressed feelings of powerlessness, guilt, and gender-based shame, suggesting that how sociocultural norms play a critical role in shaping survivors’ interpretations of their experiences. Several girls, for example, questioned the value of their gender identity or internalized the belief that being female made them inherently vulnerable. These reflections suggests that how patriarchal ideologies and societal expectations may shape often impose a disproportionate burden on girls, forcing them to navigate both the trauma of abuse and the stigma attached to being a victim.

The perception of perpetrators as familiar, trusted figures such as relatives, teachers, or community members further complicated the adolescents’ emotional responses. These betrayals disrupted not only their immediate sense of safety but also their broader ability to trust others, particularly within familial or social environments that prioritized harmony and reputation over justice. Elliott et al. (37) mentioned nearly half the offenders had no bad feelings about sexually abusing children. The emotional dissonance was heightened when abusers maintained a public facade of normalcy, behaving as though nothing had occurred. This appeared to leave participants feeling isolated and invalidated, fostering confusion about morality. Such experiences are consistent with betrayal trauma theory, which explains how abuse by trusted figures can distort children’s perception of reality and silence their attempts at resistance.

Participants also demonstrated a critical awareness of how social institutions especially schools can function as either sites of neglect or vehicles of change. Participants described schools as potentially important spaces that should actively contribute to CSA prevention through the integration of abuse awareness in curricula, the cultivation of teacher-student trust, and the establishment of clear accountability mechanisms. Their reflections emphasized the need for schools to move beyond academic instruction and embrace a more holistic, emotionally responsive approach to education. Few adolescents advocated for age-appropriate sex education and emphasized the importance of co-educational environments in fostering empathy and mutual respect, suggesting that such structural changes could normalize conversations about boundaries, consent, and bodily autonomy.

It is essential to recognize the types of abuse presented in **Table 2**. In the findings, only the least severe forms of abuse, as classified by Russell’s hierarchy of abuse severity, have been discussed. However, when examining the thematic findings, it becomes evident that the children expressed significant emotional vulnerability, described their efforts to cope with the abuse, and, in some cases, demonstrated resilience. These reactions were observed even among those who experienced the least serious forms of sexual abuse. This highlights the profound psychological impact of CSA, regardless of its perceived severity. Moreover, since these experiences pertain to adolescents who have undergone relatively less severe abuse, the intensity of their emotional and psychological responses underscores the need for early intervention and support mechanisms.

Overall, this study underscores that adolescent narratives of CSA are not only accounts of individual trauma but also reflections of broader cultural, familial, and institutional failures. The adolescents’ voices offer powerful insights into how abuse is experienced, resisted, and remembered and how healing is shaped by the responses of those around them. This indicate the value of early support in how society conceptualizes child protection, emphasizing the importance of early education, empathetic adult responses, and structural interventions. By centering the voices of survivors and recognizing the interplay of psychological, social, and cultural factors in their experiences, we move closer to developing more effective, just, and compassionate systems of support and prevention.

### 5.1 Limitations

Several limitations of the study should be acknowledged. The study involved a small sample of seven adolescent girls, which enabled an in-depth exploration of their lived experiences but limited the range of perspectives represented. The study was also conducted within a single district in Tamil Nadu; therefore, the findings may reflect the specific sociocultural and institutional context of that setting and may not be directly transferable to other regions.

The inclusion of only female adolescents limits the representation of experiences among male adolescents and children with other gender identities. Participation was voluntary, which may have resulted in self-selection bias, as adolescents who were more willing or able to discuss their experiences may have been more likely to participate, while those who were fearful, unwilling, or subject to greater social restrictions may have been underrepresented.

The study relied on participants’ retrospective accounts of their experiences, making the findings potentially subject to recall bias, particularly where considerable time had elapsed since the abuse. Given the sensitive and socially stigmatized nature of child sexual abuse, participants may also have withheld information or presented their experiences in ways they perceived as socially acceptable, creating the possibility of social desirability bias.

The findings should not be interpreted as statistically generalizable to all children who have experienced child sexual abuse. Instead, they provide context-specific insights into how adolescent girls understood and navigated their experiences within their particular sociocultural environment. The findings may nevertheless offer useful insights for understanding similar contexts and for informing further research, practice, and interventions.

## 6 Conclusion

This study offers a nuanced exploration of how adolescent girls experience and navigates child sexual abuse, suggesting that disclosure is not a single act but a continuum shaped by emotional readiness, developmental stage, and sociocultural context. The narratives reflect a complex interplay between internal processing and external expression, with some participants confidently articulating their experiences and others struggling to find language due to fear, shame, or limited awareness. These variations highlight the critical role of supportive environments in enabling or silencing disclosure, and how deeply embedded patriarchal norms often contribute to feelings of guilt, vulnerability, and self-blame among survivors.

Despite these challenges, the participants also described moments of resilience and a strong sense of agency, particularly in their vision of schools as potential spaces for prevention, support, and empowerment. Their reflections point to the need for systemic interventions that extend beyond individual responses integrating education, teacher training, and structural accountability within school settings. By centering the voice of survivors, this study shows that some adolescents described agency, resilience and ideas for support.

## Data Availability

The raw data supporting the conclusions of this article will be made available by the authors, without undue reservation.
