# Supplementary material for: Lived experiences of adolescent girls following child sexual abuse: disclosure, coping, and perceived school support
Source: Front Psychiatry. 2026 Oct 22;17:1895004. doi: 10.3389/fpsyt.2026.1895004 (PMC13736615; doi:10.3389/fpsyt.2026.1895004)
Supplement: Supplementary file 1 [file DataSheet1.docx]

# PART 1 - INFORMATION FOR PARTICIPANTS OF THE STUDY

**Institution Name:** Vellore Institute of Technology, Vellore

- **Title of the project** – “Socio-cultural influences on the psychological responses of children with experiences of child sexual abuse”
- **Name of the investigator/guide** – Dr. J. Balamurugan
- **Purpose of this project/study** - The aim of the study is to investigate the psychosocial consequences of the children who have experienced child sexual abuse. It employs a pragmatic approach and uses Mixed methods research design.
- **Procedure/methods of the study** - The target population is 14 –18year-olds in classes 9–12. Data collecting will involve these steps: First, the selected schools will receive an orientation session on child sexual abuse and its consequences. Second, the students will fill out a questionnaire to measure their exposure and awareness. Third, the researchers will interview a subset of participants who have reported experiencing child sexual abuse and volunteered to participate. Data will thereafter be collected. The data will be examined using quantitative and qualitative methods, including a standardized questionnaire and semi-structured interview.
- **Expected duration of the subject participation** – 30 - 45 mins
- **The benefits to be expected from the research to the participant or to others and the post-trial responsibilities of the investigator** - A significant number of teenagers experience child sexual abuse. In the future, this study will be beneficial for educational institutions and governmental organizations to formulate preventive initiatives targeting similar occurrences.
- **Any risks expected from the study to the participant** – Yes, there will be a minimal psychological risk as they will be sharing their experiences with the researcher. The NGO, CWC, school management, and other appropriate channels will manage the risk of identifying the perpetrator, in accordance with the scenario's requirements.
- **Maintenance of confidentiality of records** – Researchers will maintain the confidentiality of the data and utilize it exclusively for academic objectives. Only the PI and Co-PI will access each piece of data from the sample, assigning it a specific code. This will ensure the safety of the data.
- **Provision of free treatment for research related injury** – Yes
- **Compensation of the participants not only for disability or death resulting from such injury but also for unforeseeable risks** – Yes, Psychological support will be provided.
- **Freedom to withdraw from the study at any time during the study period without the loss of benefits that the participant would otherwise be entitled** - Yes
- Possible current and future uses of the biological material and of the data to be generated from the research and if the material is likely to be used for secondary purposes or would be shared with others, this should be mentioned – NA
- Address and telephone number of the investigator and co-investigator/guide

Guide:	Co- Principal Investigator:

Dr. J. Balamurugan	Ms. Silvia SV

Associate Professor,	Research Scholar

School of Social sciences & Languages	School of Social sciences & Languages

VIT, Vellore	VIT, Vellore

For ethical related queries, contact:

Prof. Pragasam Viswanathan,

Member Secretary,

Institutional Ethics Committee on Human Studies (IECH),

Vellore Institute of Technology (VIT), Vellore - 632014,

Tamil Nadu, India

# PART 2 – PARENT/GUARDIAN CONSENT FORM

**Parent’s name:	Address:**

**Title of the project**: “Socio-cultural influences on the psychological responses of children with experiences of child sexual abuse”

The details of the study have been provided to my child in writing and explained to my child in his/her own language. I confirm that my child has understood the above study and had the opportunity to ask questions. I understand that my child participation in the study is voluntary and that my child is free to withdraw at any time, without giving any reason, without the medical care that will normally be provided by the investigating team. I agree not to restrict the use of any data or results that arise from this study provided such a use is only for scientific purpose(s). I have been given an information sheet giving details of the study. I fully consent to participate my child in the above study.

Signature of the Participant:	Date:

Signature of the Witness:	Date:

Signature of the Investigator:	Date:

# PART 2 - PARTICIPANT ASSENT FORM

**Participant’s name:	Address:**

**Title of the project**: “Socio-cultural influences on the psychological responses of children with experiences of child sexual abuse”

The details of the study have been provided to me in writing and explained to me in my own language. I confirm that I have understood the above study and had the opportunity to ask questions. I understand that my participation in the study is voluntary and that I am free to withdraw at any time, without giving any reason, without the medical care that will normally be provided by the investigating team. I agree not to restrict the use of any data or results that arise from this study provided such a use is only for scientific purpose(s). I have been given an information sheet giving details of the study. I fully consent to participate in the above study.

Signature of the Participant:	Date:

Signature of the Witness:	Date:

Signature of the Investigator:	Date:
