# Supplementary material for: Lived experiences of adolescent girls following child sexual abuse: disclosure, coping, and perceived school support
Source: Front Psychiatry. 2026 Oct 22;17:1895004. doi: 10.3389/fpsyt.2026.1895004 (PMC13736615; doi:10.3389/fpsyt.2026.1895004)
Supplement: Supplementary file 2 [file DataSheet2.docx]

**பிரிவு – 1 பங்கேற்பாளர் தகவலுக்கான மாதிரி தாள்**

**ஆராய்ச்சி நிறுவனத்தின் பெயர்:** வேலூர் தொழில்நுட்ப நிறுவனம், வேலூர்

**ஆய்வின் தலைப்பு:** “பள்ளி சூழலில் வளரிளம் பருவத்தினரிடையே குழந்தை பாலியல் வன்முறை மற்றும் மன நலம் ஆகியவற்றுக்கு இடையேயான உறவைப் புரிந்துகொள்வது”

**ஆராய்ச்சியின் நோக்கம்:** பள்ளிகளில் இளம் பருவத்தினரின் குழந்தை பாலியல் வன்முறையின் அனுபவங்களை உளவியல் ரீதியாக ஆராய்வதை இந்த ஆய்வு நோக்கமாகக் கொண்டுள்ளது.

**ஆய்வின் நடைமுறைகள்:** ஆராய்ச்சியின் நோக்கமாக, சில கேள்விகள் குழந்தைகளிடம் கேட்கப்படும். அந்த கேள்விகள் 30 - 45 நிமிடங்கள் வரையில் பதிலளிக்கும் வகையில் அமைந்திருக்கும். 9-12 வகுப்புகளில் 14-18 வயதுடையவர்களை ஆய்வுக்கு எடுத்துக்கொள்ளப்படும். தரவு சேகரிப்பு பின்வரும் படிகளை உள்ளடக்கும்: முதலில், தேர்ந்தெடுக்கப்பட்ட பள்ளிகள் குழந்தை பாலியல் வன்முறை மற்றும் அதன் விளைவுகள் பற்றிய நோக்குநிலை அமர்வைப் பெறும். இரண்டாவதாக, மாணவர்கள் தங்கள் வெளிப்பாடு மற்றும் விழிப்புணர்வை அளவிட ஒரு கேள்வித்தாளை நிரப்புவார்கள். மூன்றாவதாக, குழந்தைகள் பாலியல் வன்முறையை அனுபவித்ததாகப் புகாரளித்த மற்றும் பங்கேற்க முன்வந்த பங்கேற்பாளர்களை ஆராய்ச்சியாளர்கள் நேர்காணல் செய்வார்கள். அதன் பிறகு தரவுகள் சேகரிக்கப்படும். தரப்படுத்தப்பட்ட கேள்வித்தாள் மற்றும் அரை-கட்டமைக்கப்பட்ட நேர்காணல் உள்ளிட்ட அளவு மற்றும் தரமான முறைகளைப் பயன்படுத்தி தரவு ஆய்வு செய்யப்படும்.

**ஆராய்ச்சியில் பங்கேற்பதிலுள்ள ஆபத்து:** உள்ளது, அவர்கள் தங்கள் அனுபவங்களை ஆராய்ச்சியாளருடன் பகிர்ந்து கொள்வதால், குறைந்தபட்ச உளவியல் ஆபத்து இருக்கும். NGO, CWC, பள்ளி நிர்வாகம் மற்றும் பிற பொருத்தமான சேனல்கள் சூழ்நிலையின் தேவைகளுக்கு ஏற்ப, குற்றவாளியை அடையாளம் காணும் அபாயத்தை நிர்வகிக்கும்.

**ஆராய்ச்சியில் பங்கேற்பதிலுள்ள நன்மைகள்:** குழந்தைகளின் பாலியல் வன்முறையால் எத்தனையோ வளறிளம் பருவத்தினர் பாதிக்கப்பட்டுள்ளனர். எதிர்காலத்தில், இது போன்ற நிகழ்வுகளை தடுக்க பள்ளிகள் அல்லது அரசு சில திட்டங்களை உருவாக்கவும் இந்த ஆய்வு உதவியாக இருக்கும்.

**பங்கேற்பாளர்களுக்கு இத்தகைய காயத்தால் ஏற்படும் இயலாமை அல்லது இறப்புக்கு மட்டுமின்றி எதிர்பாராத அபாயங்களுக்கும் இழப்பீடு:** ஆம், உளவியல் ஆதரவு வழங்கப்படும்.

**இரகசியத்தன்மை:** ஆராய்ச்சியாளர்கள் தரவின் ரகசியத்தன்மையைப் பேணுவார்கள் மற்றும் கல்வி நோக்கங்களுக்காக பிரத்தியேகமாக அதைப் பயன்படுத்துவார்கள். PI மற்றும் Co-PI மட்டுமே மாதிரியிலிருந்து ஒவ்வொரு தரவையும் அணுகும், அதற்கு ஒரு குறிப்பிட்ட குறியீட்டை ஒதுக்கும். இது தரவுகளின் பாதுகாப்பை உறுதி செய்யும்.

**பங்கேற்பாளரின் உரிமைகள்:** பங்கேற்பாளர்கள் எப்போது வேண்டுமானாலும் ஆராய்ச்சியிலிருந்து விடை பெறலாம். ஆராய்ச்சியில் கேட்கப்படும் கேள்விகள் பங்கேற்பாளரை வருத்தமடையவோ அல்லது பதில்கூற விருப்பமில்லை என்று நினைத்தால் குழந்தைகள் தாராளமாக ஆராய்ச்சியில் இருந்து விலகிக்கொள்ள முழு உரிமை உண்டு என்பதை தெரிவித்துக கொள்கிறோம்.

**ஆய்வுத் தொடர்பான கேள்விகள் மற்றும் சந்தேகங்களுக்கு**:

**முதன்மை ஆய்வாளர்:** முனைவர். ஜெ. பாலமுருகன்

**தொடர்புக்கொள்ள வேண்டிய முகவரி:** சமூக அறிவியல் மற்றும் மொழிகள் துறை, வேலூர் தொழில்நுட்ப நிறுவனம், வேலூர்,

**ஆய்வாளர்:** சில்வியா சிவி

**தொடர்புக்கொள்ள வேண்டிய முகவரி:** சமூக அறிவியல் மற்றும் மொழிகள் துறை, வேலூர் தொழில்நுட்ப நிறுவனம், வேலூர்,

**உறுப்பின செயலாளர்:** பேராசிரியர். பிரகாசம் விஸ்வநாதன்

**தொடர்புக்கொள்ள வேண்டிய முகவரி:** மனித ஆய்வுகளுக்கான நிறுவன நெறிமுறைக் குழு (IECH), சமூக அறிவியல் மற்றும் மொழிகள் துறை, வேலூர் தொழில்நுட்ப நிறுவனம், வேலூர்,

**பிரிவு 2 –பெற்றோர்/பாதுகாவலர் ஒப்புதல் படிவம்**

**ஒப்புதல் படிவம்**

பெற்றோரின் பெயர்:	முகவரி:

ஆய்வின் தலைப்பு: **“பள்ளி சூழலில் வளரிளம் பருவத்தினரிடையே குழந்தை பாலியல் வன்முறை மற்றும் மன நலம் ஆகியவற்றுக்கு இடையேயான உறவைப் புரிந்துகொள்வது”**

"மேற்கூறிய தகவலை நான் படித்திருக்கிறேன், அல்லது அது எனக்கு வாசிக்கப்பட்டது. என்னிடம் அதைப்பற்றி கேள்விகள் கேட்க வாய்ப்பு மற்றும் நான் கேட்ட கேள்விகளுக்கு என் திருப்திக்கு பதிலளித்தார். என் குழந்தையை சேர்க்க நான் தானாக முன்வந்து என் சம்மதத்தை அளிக்கிறேன். இந்த ஆய்வில் பங்கேற்று, படிப்பிலிருந்து விலக எனக்கு உரிமை உண்டு என்பதை புரிந்துக்கொள்கிறேன். எந்த நேரத்திலும் படிப்பிலோ அல்லது எனது மேலும் மருத்துவ சேவையை எந்த விதத்திலும் பாதிக்காமல்.”

● எனது குழந்தை இந்த ஆய்வில் ஒரு பங்கேற்பாளராக சேர்க்கப்படுவதற்கு எனது ஒப்புதலை அளிக்கிறேன்.

● இந்த ஆய்வில் என் குழந்தை வலுக்கட்டாயமாக பங்கேற்க வேண்டும் என்ற அவசியம் இல்லை என்ற தனது குழந்தையின் உரிமையையும் நான் அறிவேன்.

● இந்த ஆய்வில் என் குழந்தை பங்கேற்பதற்கு நான் ஒப்புக்கொள்கிறேன்.

பெற்றோரின் கையொப்பம்:	தேதி:

ஆதாரம் கையொப்பம்:	தேதி:

ஆய்வாளரின் கையொப்பம்:	தேதி:

**பிரிவு 2 - பங்கேற்பாளர் ஒப்புதல் படிவம்**

பங்கேற்பாளரின் பெயர்:	முகவரி:

ஆய்வின் தலைப்பு: **“பள்ளி சூழலில் வளரிளம் பருவத்தினரிடையே குழந்தை பாலியல் வன்முறை மற்றும் மன நலம் ஆகியவற்றுக்கு இடையேயான உறவைப் புரிந்துகொள்வது”**

"மேற்கூறிய தகவலை நான் படித்திருக்கிறேன், அல்லது அது எனக்கு வாசிக்கப்பட்டது. என்னிடம் அதைப்பற்றி கேள்விகள் கேட்க வாய்ப்பு மற்றும் நான் கேட்ட கேள்விகளுக்கு என் திருப்திக்கு பதிலளித்தார். என் குழந்தையை சேர்க்க நான் தானாக முன்வந்து என் சம்மதத்தை அளிக்கிறேன். இந்த ஆய்வில் பங்கேற்று, படிப்பிலிருந்து விலக எனக்கு உரிமை உண்டு என்பதை புரிந்துக்கொள்கிறேன். எந்த நேரத்திலும் படிப்பிலோ அல்லது எனது மேலும் மருத்துவ சேவையை எந்த விதத்திலும் பாதிக்காமல்.”

● நான் இந்த ஆய்வில் ஒரு பங்கேற்பாளராக சேர்க்கப்படுவதற்கு எனது ஒப்புதலை அளிக்கிறேன்.

● இந்த ஆய்வில் நான் வலுக்கட்டாயமாக பங்கேற்க வேண்டும் என்ற அவசியம் இல்லை என்ற எனது உரிமையையும் நான் அறிவேன்.

● இந்த ஆய்வில் பங்கேற்பதற்கு நான் ஒப்புக்கொள்கிறேன்.

பங்கேற்பாளரின் கையொப்பம்:	தேதி:

ஆதாரம் கையொப்பம்:	தேதி:

ஆய்வாளரின் கையொப்பம்:	தேதி:
